# A study on the effect of fingerprints in a wet system

**DOI:** 10.1038/s41598-019-51694-9

**Published:** 2019-11-12

**Authors:** Donghyun Kim, Dongwon Yun

**Affiliations:** Daegu Gyeongbuk Institute of Science & Technology(DGIST), Department of Robotics Engineering, Daegu, Republic of Korea, 333 Techno jungang-daero, Hyeonpung-eup, Dalseong-gun, Daegu, 42988 South Korea

**Keywords:** Engineering, Physics

## Abstract

In this paper, we study the influence of the fingerprint and sweat on the fingerprint on the friction between the hand and an object. When sweat contacts a finger or an object, it is sometimes easy to pick up the object. In particular, we can see this phenomenon when grasping a thin object such as paper and vinyl. The reason for this phenomenon is the increase of friction force, and this paper physically analyzes this natural phenomenon. To this end, we investigate the cause of the friction force between a solid and liquid to calculate the friction force when water is present within the fingerprint. To support the theoretical analysis, we conduct experiments to measure the friction force by making a finger-shaped silicon specimen. By comparing the theoretical and experimental results, we defined the change of friction force if there was water in the fingerprint. Through this study, it is possible to analyze the role of the fingerprint and sweat on the finger, and thereby explain the friction change depending on the amount of sweat.

## Introduction

The parts of the human body have their respective roles. The hands serve to hold objects, the feet to walk, the eyes to see. Eyebrows prevent foreign substances from entering the eye and hair plays a role in protecting the skull. In this study, we examined the role of fingerprints. While fingerprints are used to identify individuals^1^, this is not their fundamental role from a biological perspective. In this study, we inferred that fingerprint helps humans hold objects. If we can show that the fingerprint facilitates the friction force between the object and the hand, then we can potentially exploit control over the friction force in many different fields. Therefore, we studied the effect of fingerprints on the change of friction force.

In general, people think that fingerprints increase the friction between objects and fingers. However, previous studies have shown that fingerprints of human hands play a role in lowering friction^2^. Some studies have presented results showing that it is possible to hold heavier objects when the hand has no fingerprints^3^. G. Chimeta *et al*. concluded that the presence of fingerprints reduces the frictional force through an experiment to measure the friction force with and without fingerprints by making artificial silicon fingers^4^. However, in other studies, it was concluded that fingerprints can increase friction force, and they can control friction force by changing the contact area according to the angle of the human finger^5,6^. By controlling friction force using fingerprints, some studies proved that fingerprints can help to grasp^7–9^. Although the researchers have continued to the friction of fingers, the majority of research on this phenomenon has been carried out through experiments rather than theoretical analysis. Researchers have studied how fingerprints change friction force through experiments in molecular units and found that the fingerprints play a major role in controlling the friction force^10^. Generally, the friction force only depends on the constituents of the two objects and the shape of the object cannot change the friction force. However, when the contact area between objects is larger, the frictional force becomes greater^6^. This means that there is a correlation between force and contact area, and many papers have proved this experimentally.

As the contact area increases, the friction force tends to increase accordingly. However, some studies have concluded that the friction force can change even when the contact area is constant^11–13^. In the case of high humidity, the frictional force between an object and the hand becomes higher. Empirically, if we have water on our hands, we can hold an object well. Although there is no way to directly increase the friction force only by the fingerprint itself, if the water is in the fingerprint, the friction force can change^14^. The relationship between water and friction is not a simple linear relationship. Previous researchers revealed through an experiment a tendency of the friction force to increase up to a certain point, and decrease thereafter like a quadratic function^8^.

Furthermore, the possibility of increasing friction in many materials other than the hand using water has been reported in many studies. We can calculate the friction force precisely using a two-dimensional molecular structure instead of the complex actual form^15,16^. The cause of the increase of friction force by water is the surface tension^17^. The force of this surface tension is large enough that a snail can move by controlling the mucus^18^. We call the force generated by the surface tension adhesive force. The adhesive force can affect the motion of an object. Some researchers have made movement by determining the material and shape of a surface to control adhesive force^19^. Additional friction force comes from surface tension, and researchers derived equations of additional friction force using experimental result^20^. Previous studies have proved through experimental analyses that the principle underlying the surface tension is intermolecular attraction^21,22^. By using the principle of surface tension, it is possible to calculate the friction force when there is a water bridge on a rough surface^23^.

Many studies have suggested the possibility of controlling the friction force by water and numerous studies have explained this phenomenon by experimental methods. Also, based on these principles, we can see that the fingers work more strongly in high humidity environments. There is a paper that experimentally calculates the additional friction force and formulates the additional force through the numerical analysis^20^. However, it was not possible to derive the cause of the additional friction force and the theoretical formula accordingly. In this paper, we have analyzed the relationship between the fingerprint geometry and the amount of water, and examined the role of the fingerprint and sweat in gripping objects with human fingers.

**Figure 1:**
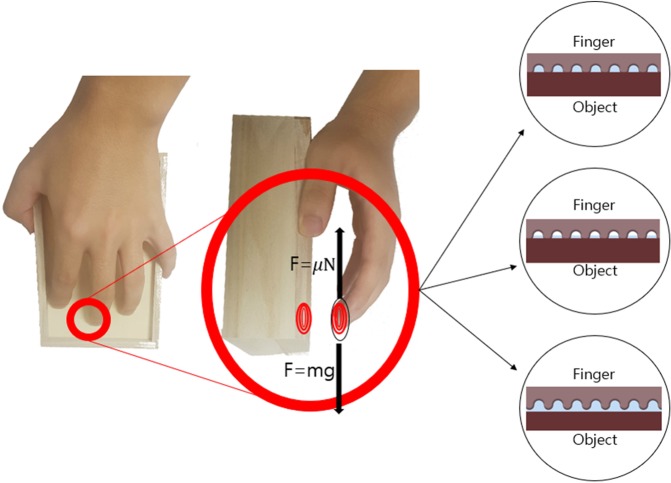
Measuring friction force between hand and object when water is in fingerprint.

We have experimentally and theoretically investigated how the friction force works in each case depending on the amount of water around the fingerprint. It is easy to judge whether the change of the friction force is meaningful from the magnitude of the friction force. This could be exploited in various fields if analyzing the change of friction force is possible. In particular, we can create a mechanism to control the force applied to an object using friction force. Figure 1 illustrates this paper’s concept.

Basically, the factors that influence the increase of friction due to water are the same as those that generate friction force. There are various factors such as intermolecular force and electromagnetic force. The sum of these values is implied in the concept of surface energy. This energy is measured largely between solids and solids, and it depends on the force acting on the interface between the objects. It works like vertical force. The surface force between a solid and a liquid has a different tendency. The most powerful force between a solid and a liquid is the intermolecular force. The water molecules consist of hydrogen bonding and thus can polarize to the surrounding surfaces. By this coupling, an attraction between water and the surface acts to attract each other and thus water can affect the contact between objects, which can control the friction of the surface.

There are two ways in which adhesive force can influence the friction force. One is the case where adhesive force arises due to the surface energy between the object. In the case of water between the fingerprints, the surface energy between the water and the hand causes the adhesive force to adhere to the two objects. The magnitude of this force is 0 when the object does not move, but when the object moves, the adhesive force acts in the opposite direction of the movement. Adhesive force can be determined through between the surface energy and the width of the contact surface^24–26^. In addition, it is available to calculate the additional friction force due to the amount of water.

Viscous force occurs when water comes into contact with the object. If there is more water than the volume of fingerprint, the water will spill over the fingerprint. In this paper, we assume that a thin water film will form below the finger when it moves. In this system, the viscosity that occurs during movement by the water film plays a role of friction force.

This paper derives theoretical formulas for the friction force added by the adhesive force. The next chapter covers this contents.

## Results

### Adhesive force due to water in the fingerprint

The factors affecting adhesive force are Van der Waals force and electromagnetic force. These forces appear as surface energies, which appear as constants of matter in the same environment (temperature, humidity, area). The adhesive force acts when the interfacial adhesion state changes, and we can express it through the area and the work of adhesion. Work of adhesion depends on the arrangement of the object. If an object is in the air, the work of adhesion is the interaction between the surface of the object and the air. If we assume that there is fluid around the attached objects, there will be surface energy between these two objects and the fluid. When two attached objects fall apart, a new surface energy occurs between the air and the two objects and the energy between the two existing objects disappears. The difference in this energy acts as work of adhesion. Therefore, if object i, j falls in fluid k, the value of work of adhesion appears as given in Eq. 1.

1 $${\varpi }_{ikj}={\gamma }_{ik}+{\gamma }_{jk}-{\gamma }_{ij}$$

In general, we use this equation to measure the surface energy between a solid and a solid. But we also can use it to measure the energy between a solid and a liquid. ${\gamma }_{ik}$ denotes the surface energy between i, k. To measure the surface energy between two objects, we should classify the dispersion force and polar force because these different forces act on each other. Equation 2 shows how to calculate it.

2 $${\gamma }_{ij}=\,{\gamma }_{i}+{\gamma }_{j}-2\sqrt{{\gamma }_{i}^{d}{\gamma }_{j}^{d}}-2\sqrt{{\gamma }_{i}^{p}{\gamma }_{j}^{p}}$$

${\gamma }_{i}\,$is the surface tension between air and an object i. It is expressed as the sum of ${\gamma }_{i}^{d}$ and ${\gamma }_{i}^{p}$. ${\gamma }_{i}^{d}$ and ${\gamma }_{i}^{p}$ are object i’s dispersion force and polar force. Using Eqs 1 and 2, we can obtain the work of adhesion between i, k in water. Equation 3 shows this.

3 $${\varpi }_{iVj}=2(\sqrt{{\gamma }_{i}^{d}{\gamma }_{j}^{d}}+\sqrt{{\gamma }_{i}^{p}{\gamma }_{j}^{p}})$$

Equation 3 shows how to calculate the work of adhesion between water and objects. In a fingerprint situation, two cases of energy occur: energy between the hand and water and energy between the ground and water. Equation 3 shows the way to calculate it. First, we can calculate the work of adhesion between the water and hand (${\varpi }_{SL}$) and between water and object(${\varpi }_{LU}$). ${\varpi }_{SL}$ is the result of Eq. 3 when material i is water, and material j is silicon. ${\varpi }_{LU}$ is the result of Eq. 3 when material i is water, and material j is plastic, the material of the ground plate. Equation 4 then shows the way to determine the adhesive force.

4 $${F}_{ad}={\alpha }_{adh}({\varpi }_{SL}+{\varpi }_{LU})+{\beta }_{adh}{\varpi }_{SL}$$

${\alpha }_{adh}({\varpi }_{SL}+{\varpi }_{LU})$ shows the adhesive force at the top and bottom of the fingerprint. ${\beta }_{adh}{\varpi }_{SL}$ shows the force at the side of the fingerprint. We should divide force like these two factors because the surface tension interacts in parallel plates. ${\alpha }_{adh}$ and ${\beta }_{adh}$ are constants that can change according to the fingerprint’s condition like depth, length, shape.

If there is a water film between the plate and the ground, we can calculate the force from water using the law of surface tension. Its effects are similar to adhesive force, and Eq. 5 shows the adhesive force when the height of the water film is h, the contact area is A, and the surface tension between the water and plate is ${\rm{\omega }}$.

5 $${F}_{ad}=\frac{2\omega A}{h}$$

Equation 5 shows that the adhesive force and distance between the plate and ground have an inverse relation. Using this phenomenon, we can calculate ${\alpha }_{adh}$ and ${\beta }_{adh}$ when we know the shape of the fingerprint. It is easy to obtain this force when the shape of the fingerprint is simple like a rectangular parallelepiped. Considering the number and size of fingerprints, we can obtain Eq. 6 when the shape of the fingerprint is a rectangular parallelepiped. d is the width of the fingerprint, n is the number of fingerprints, and B is the surface area of the fingerprint’s side face.

6 $${F}_{ad}=\frac{A}{h}({\varpi }_{SL}+{\varpi }_{LU})+\frac{nB}{d}{\varpi }_{SL}$$

### Viscous force due to water in the fingerprint

The tendency of the frictional force when the water overflows the fingerprint is completely The tendency of the frictional force when the water overflows the fingerprint is completely different from non-overflow. In this case, viscous force can work like friction force. We will assume that the water in the fingerprint does not flow and moves with the fingerprints and fingers. A water film exists under this water, and we can assume that this film is a Newtonian fluid, and this film creates a force that interferes with the moment. We call this viscous force *F*_*v*_ and Eq. 7 gives the magnitude of this force.

7 $${F}_{v}=\mu A\frac{v}{h}$$

*μ* denotes viscosity, A is the contact area, *v* is velocity, and *h* is the height of the film. We can calculate adhesive force using Eq. 5 if we know the vertical force and surface tension. In this case, height is not a constant, and therefore we should calculate the force differently at the node and the valley of the hand. Assuming the area of the valley part is 1/3 of the entire finger area, Eq. 8 shows the vertical force.

8 $${F}_{T}=\sum _{Node\,part}\frac{4\sigma A}{3h}+\sum _{Valley\,part}\frac{2\sigma A}{3(h+h^{\prime} )}$$

In Eq. (8), *F*_*T*_ represents vertical force and $h^{\prime}$is the depth of the fingerprint. Equation (8) can be rewritten as a quadratic equation of h (Eq. (9)).

9 $${F}_{T}{h}^{2}+(h^{\prime} {F}_{T}-2\sigma A)h\,-\frac{4}{3}\sigma Ah^{\prime} =0$$

Hence, we can see ${F}_{v}$ in Eq. (10) $\sigma$ is the surface energy of water.

10 $${F}_{v}=\frac{2\mu A{F}_{T}}{2\sigma A-h^{\prime} {F}_{T}+\sqrt{{(h^{\prime} {F}_{T}-2\sigma A)}^{2}+\frac{16}{3}{F}_{T}\sigma Ah^{\prime} }}\nu $$

When the vertical drag is not excessive, or when the water is overflowing, the friction force by the viscosity will act, and when the vertical drag exceeds the individual level, or when the water is located only inside the fingerprint, the friction force by the adhesive force will act.

### Combination of fingerprint and water

**Figure 2:**
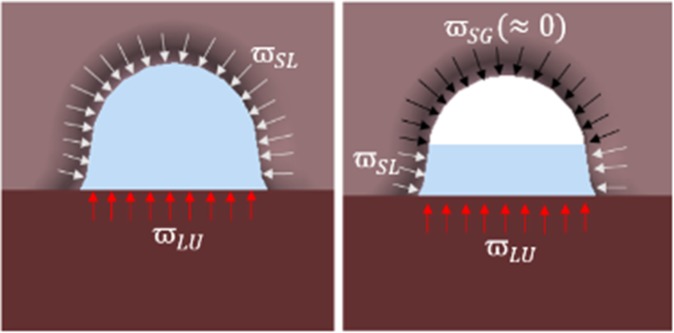
The way that adhesive force affects objects.

Adhesive force changes when the amount of water changes. ${\alpha }_{adh}$ and ${\beta }_{adh}$ then change such that the tendency of the equation changes. The surface tension of water determines the maximum height of water at which water can be attached to the ceiling portion of the fingerprint. We call this height ${h}_{l}$ and can derive three types of equations when the height of water ${h}_{w}$ changes, as given in Eqs 11–13. Figure 2 shows how the adhesive force works between the object, hand, and water.

11 $${F}_{ad}=({\alpha }_{adh}(\frac{{h}_{w}}{h})+{\beta }_{adh}){\varpi }_{SL}+{\alpha }_{adh}{\varpi }_{LU}(h\ge {h}_{w}\ge {h}_{l})$$

12 $${F}_{ad}={\beta }_{adh}(\frac{{h}_{w}}{h}){\varpi }_{SL}({h}_{l}\ge {h}_{w}\ge 0)$$

13 $${F}_{ad}=\frac{2\mu A{F}_{T}}{2\sigma A-h^{\prime} {F}_{T}+\sqrt{{(h^{\prime} {F}_{T}-2\sigma A)}^{2}+\frac{16}{3}{F}_{T}\sigma Ah^{\prime} }}\nu \,({h}_{w}\ge h)$$

Equation 11 represents the force when the water drops and thus the ceiling water disappears. Equation 12 gives the time when the water in the column disappears. Equation 13 shows the case where the water overflows to the lower part. Figure 1 shows the relationship between the amount of water in these three cases and the fingerprint size. Each figure contains a case where Eqs 11–13 occur.

### Experimental material

**Figure 3:**
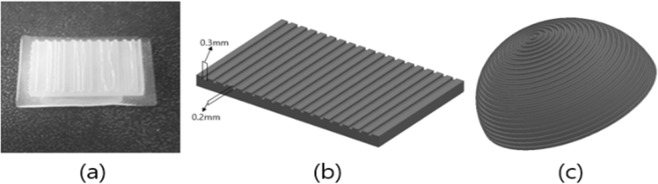
Shape of silicon finger.

We carried out experiments to verify the theory. One of the reasons that water can greatly change the friction force on the fingers is the softness of the fingers, that is, the small Young’s modulus. Using a soft material increases the contact area according to the gripping force, which can result in greater additional friction of the water. To reflect this, we used a silicon, a soft material. Figure 3(a) shows a rectangular-shaped silicon sample. The silicon is translucent with a hardness of 20. To make it, we used a plastic mold, which was filled with molding silicon. Since it is difficult to perform the experiment with an actual fingerprint, this approach makes it relatively easy to compare the result with the theoretical value. Figure 3 shows the exact size of the fingerprint. The reason why we used this size is that, in order to maximize the amount of water in the fingerprint, there should be no water loss during the experiment. In the case of real fingerprints, we thought that the silicon pads could fulfill this role faithfully, and hence we chose a size that is similar to actual fingerprint size.

**Figure 4:**
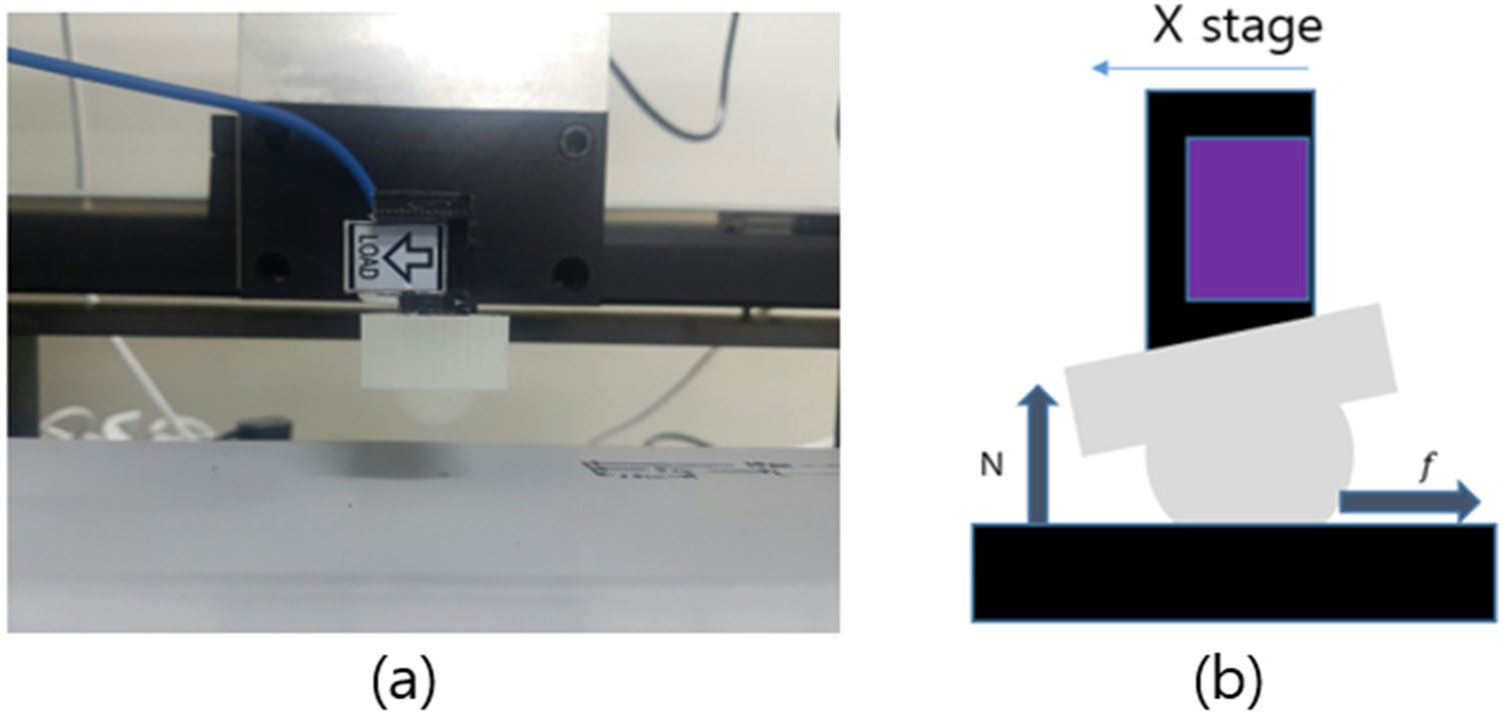
Friction force experiment setting.

In order to make use of the characteristics of the soft material, we measured the amount of additional force when the contact area changes. To this end, we made a spherical silicon finger that has a fingerprint, as shown in Fig. 3(c). We made its finger body to the hemisphere shape, and made a circular fingerprint based on the pole region of the finger body. The direction of the fingerprint was made in the normal direction of the finger body, so its shape is as similar to the human finger. We used 3D printer to create a mold of the finger. Figure 4 shows the shape of actual finger. We conducted the same experiment inserting fingerprints into spherical silicon. Using spherical silicon, it is possible to calculate the contact area according to the height, as well as the change in the additional friction force according to the area.

We used a load cell to measure the friction force^27^. The load cell is a force sensor that measures the force difference between both ends through current change. We used a load cell that can measure 3 kgf, or about 30N, and has a resolution of 0.01N. We chose it because the magnitude of the force that we need is not large but requires precise measurement. There are two forces to measure, and the directions of these two forces are orthogonal, and therefore two experiments should be conducted. In the case of soft material, the contact area changes according to the vertical force, and the friction force also changes.

Therefore, the position when measuring two forces are important, and the stage can control it. The two degree of freedom stage can adjust the position of the object precisely, and we also can use the stage when measuring the kinetic friction force. Finally, we calculated the friction force according to the change of water; because we needed a uniform surface, we used a plastic plate. Figure 4 shows the experiment setting.

### Relation between water and friction force

We conducted an experiment to obtain the friction force between the ground and silicon. It is common to obtain the friction force through the difference between the force when object does not move and the force when it moves. However, when the silicon pad moves, stiffness of the silicon pad generates the repulsive force, so it is difficult to measure the friction force. Since the friction force acts in the opposite direction of the motion, we conducted the experiment two times, changing the direction of movement. We assumed that the difference between the two measured values is twice the actual value.

**Figure 5:**
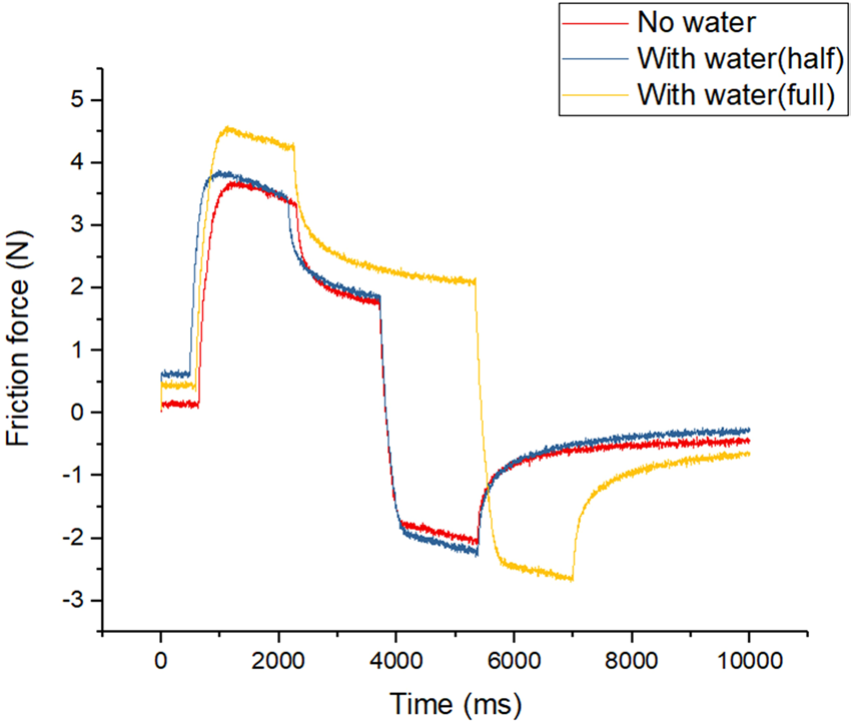
Difference of friction force according to water.

There is no way to accurately measure the amount of water in the fingerprint. We assume that the point where the friction force is maximal is the point where the fingerprint is completely filled with water. We performed three experiments (no water in fingerprint, fingerprint is full of water, fingerprint is half-filled). We used a pipette to control the amount of water. To calculate the friction force when the fingerprint is completely filled, we used 0.02 mL of water. In the half-filled case, we used 0.01 mL of water. Figure 5 shows the results of the experiment. We can see that there is a significant difference in friction force between half water and full water. The reason of this phenomena is that the size of the contact surface between the fingerprint and water changes the friction force proportionally. When water is full, the water is attached to the ceiling point of fingerprint, so the variation of the friction force become larger.

Initially there is no change in the friction force, but when the object begins to move, the friction force increases. The friction force reaches a peak and then gradually falls. After the object has stopped, friction falls but the stiffness of the object disturbs the calculation of the real friction force, and therefore the object was moved to the opposite side. In this process, the force acts on the other side and the friction force value becomes negative.

**Figure 6:**
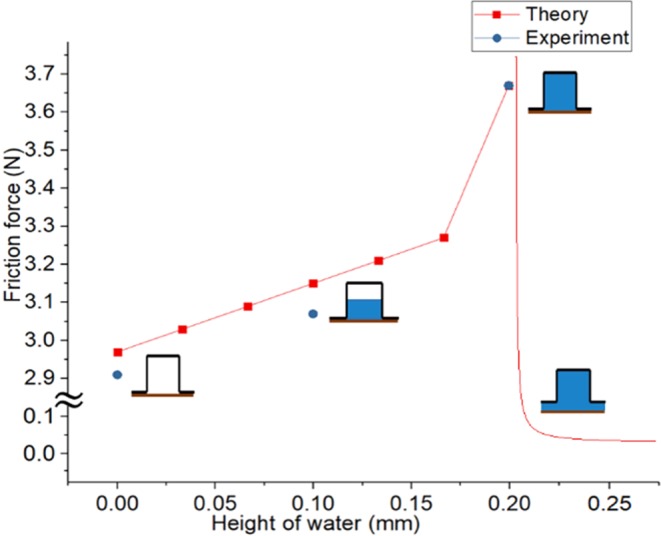
Theoretical, experimental results of friction force according to the height of water.

Figure 6 shows the theoretical and experimental results. The red line shows the theoretical results of friction force. The blue dot indicates the experimental results in the three cases. To derive the theoretical results, we used Eqs 11–13 and the equation of friction force when there is no water (..). The surface energy creates additional friction force before the water height reaches 0.2 mm, and the viscous force creates friction force when the height is greater than 0.2. ${F}_{ad}$ is the additional force and thus the real friction force is the sum of ${F}_{ad}$ and ${\mu }_{k}N$. Equation 14 shows the real friction force when water is present. We calculated the coefficient of friction and the vertical force through an additional experiment. The COF is 0.45 and the vertical force is 6.6 N. We assumed that the velocity is 1 m/s when calculating the viscous force. Table 1 shows the remaining constant values.

14 $${\rm{F}}=\mu N+\frac{A}{h}({\varpi }_{SL}+{\varpi }_{LU})+\frac{nB}{d}{\varpi }_{SL}$$

**Table 1:** Constant values of equation at rectangular form.

Size of fingerprint and finger					Work of adhesion	
h	A	d	n	B	${{\boldsymbol{\varpi }}}_{{\bf{S}}{\bf{L}}}$	${{\boldsymbol{\varpi }}}_{{\bf{L}}{\bf{U}}}$
0.2 mm	2 cm^2^	0.3 mm	20	0.4 cm^2^	0.15 N/m	0.15 N/m

The next section shows the relationship between the contact area and friction force.

### Relation between contact area and friction force

**Figure 7:**
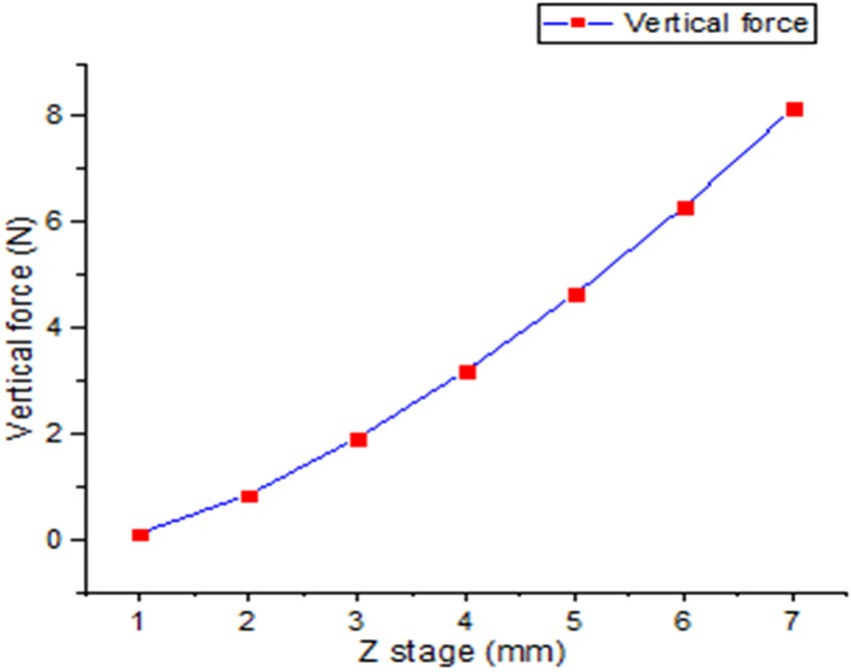
Measurements of vertical force changing Z stage.

We performed this experiment to confirm that the water can increase the friction force more when there is large contact area compared to the case of small contact area. Making silicon finger as presented in Fig. 3(c) is advantageous to change the contact area. We can change the contact area by changing the vertical force. The vertical force changes when the Z stage changes. Figure 7 shows the relationship between the Z stage and vertical force. Theoretically, the contact area is proportional to ${w}^{2/3}$^20^.

$w$ is the vertical force. Equation 15 shows the equation to derive the contact area and vertical force^20^.

15 $${\rm{A}}={\rm{\pi }}{[\frac{3{R}_{s}{(1-v)}^{2}}{4E}]}^{2/3}\,{w}^{2/3}$$

**Figure 8:**
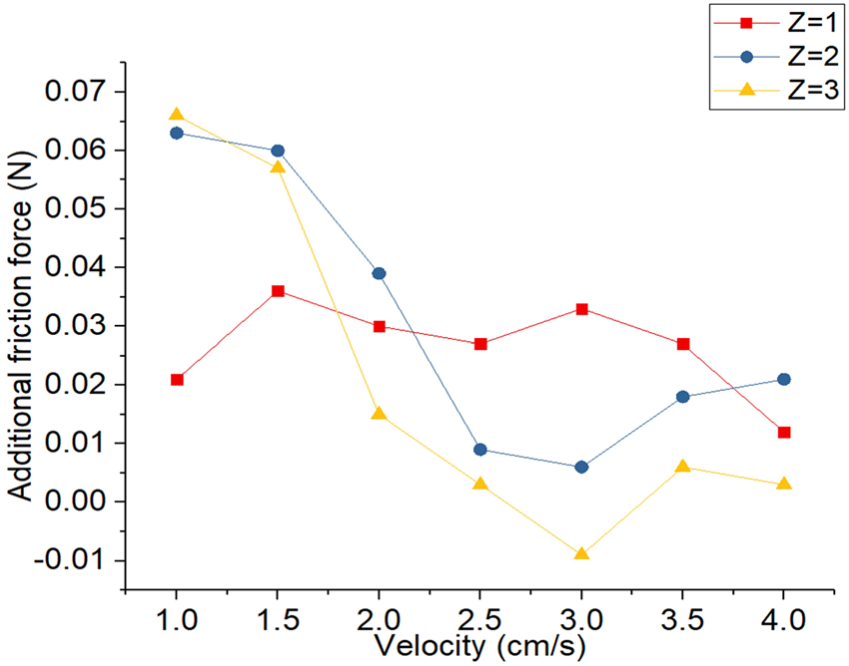
Relation between vertical force and contact area.

The contact area is proportional to *w*^2/3^ and we can represent constant values using Young’s modulus (E), Poisson’s ratio (*v*), and the radius of the finger (*R*_*s*_)^28^. We can calculate this through the experiment. Equation 15 and Fig. 7 show the correlation relation between them, and Fig. 8 also shows the relation.

16 $${\rm{A}}=15{w}^{2/3}$$

**Figure 9:**
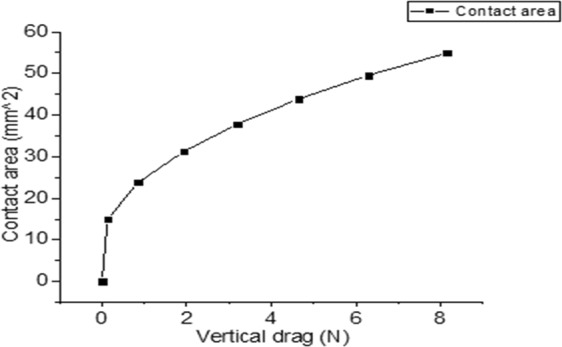
Addition force when finger speed changes.

Due to the nature of the silicon, the stiffness is inevitably large. In general, the stiffness according to the speed does not change. Therefore, it is necessary to measure the friction force regardless of speed. However, it is difficult to measure the friction force accurately by moving the silicon quickly because the restoration speed of the silicon is slow. Figure 9 shows the additional force due to water at different speeds and we can see that the portion where the friction force varies depending on the constant area is the low speed portion. Therefore, we set the difference of the friction force at the lowest speed as the actual experimental value.

At low velocity data is the most reliable. We can compare this data with Eq. 6. Unlike the rectangular form fingerprint, the length of the fingerprint is not constant and the area of the side space is different. Since the area is proportional to the diameter of the finger that forms the fingerprint, we can calculate length of fingerprint(B). Table 2 shows the constant values for the spherical finger.

**Table 2:** Constant values of equation at spherical form.

	Size of fingerprint and finger					Work of adhesion	
	h	A	d	n	B	${{\boldsymbol{\varpi }}}_{{\boldsymbol{S}}{\boldsymbol{L}}}$	${{\boldsymbol{\varpi }}}_{{\boldsymbol{L}}{\boldsymbol{U}}}$
Z = 1	0.3 mm	9.32 mm^2^	0.3 mm	6	2.1 mm^2^	0.15 N/m	0.15 N/m
Z = 2	0.3 mm	17.66 mm^2^	0.3 mm	8	3.91 mm^2^	0.15 N/m	0.15 N/m
Z = 3	0.3 mm	25.02 mm^2^	0.3 mm	12	4.12 mm^2^	0.15 N/m	0.15 N/m

**Figure 10:**
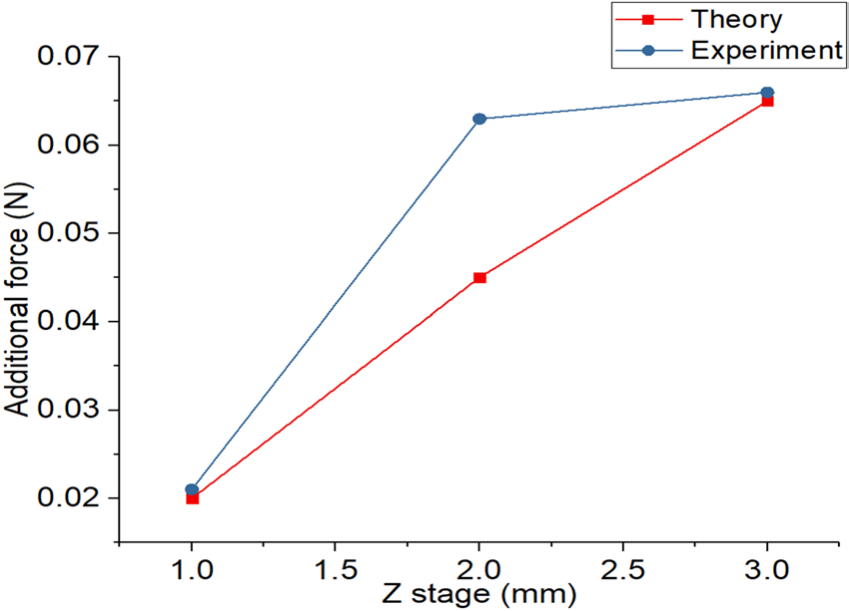
Theoretical, experimental results of friction force according to the contact area.

Figure 10 shows the theoretical results of the friction force when the Z stage changes and the experimental results.

## Discussion

Experiments with a rectangular fingerprint proved the hypothesis that the adhesion of water inside the fingerprint will increase the friction force. We can see that the surface force of water can change the friction force between two objects. A rectangular fingerprint experiment also shows that the additional force follows Eqs 11–13 and this means that we can control the friction force precisely. Theoretically and experimentally, we can see that this change is significant with respect to the act of picking up real objects. The surface tension that acts between two parallel plates makes this additional force, and the force is proportional to the contact area and is inversely proportional to the distance between the two plates. Also, the proportional constant is a specific constant determined between the object and the object, and surface tension can determine this value.

From the spherical silicon experiment, we can see that the contact area increases, and the friction force also increases when there is a wet system. The reason for the error is the stiffness of silicon. The stiffness increases when the height of the object increases. A spherical finger has considerable height, and hence there is large error.

In future studies the authors will carry out additional experiments for more accurate measurement of friction forces. By processing the shape of the fingerprint more precisely and accurately measuring the amount of water in the fingerprint, we can conduct experiments that can measure the friction force more accurately. The results presented in this paper include the approximate tendency of the theory, but in order to put the technology into practical use we must measure the amount of water and the magnitude of the force. If this is possible, this technology will greatly contribute to the development of science and technology.
